# Segmentation for Multi-Rock Types on Digital Outcrop Photographs Using Deep Learning Techniques

**DOI:** 10.3390/s22218086

**Published:** 2022-10-22

**Authors:** Owais A. Malik, Idrus Puasa, Daphne Teck Ching Lai

**Affiliations:** 1 School of Digital Science, Universiti Brunei Darussalam, Brunei Darussalam, Gadong BE1410, Brunei; 2 Institute of Applied Data Analytics, Universiti Brunei Darussalam, Brunei Darussalam, Gadong BE1410, Brunei; 3 Brunei Shell Petroleum, Brunei Darussalam, Panaga KB2933, Brunei

**Keywords:** sedimentology, segmentation, deep learning, multi-rock types, digital outcrop

## Abstract

The basic identification and classification of sedimentary rocks into sandstone and mudstone are important in the study of sedimentology and they are executed by a sedimentologist. However, such manual activity involves countless hours of observation and data collection prior to any interpretation. When such activity is conducted in the field as part of an outcrop study, the sedimentologist is likely to be exposed to challenging conditions such as the weather and their accessibility to the outcrops. This study uses high-resolution photographs which are acquired from a sedimentological study to test an alternative basic multi-rock identification through machine learning. While existing studies have effectively applied deep learning techniques to classify the rock types in field rock images, their approaches only handle a single rock-type classification per image. One study applied deep learning techniques to classify multi-rock types in each image; however, the test was performed on artificially overlaid images of different rock types in a test sample and not of naturally occurring rock surfaces of multiple rock types. To the best of our knowledge, no study has applied semantic segmentation to solve the multi-rock classification problem using digital photographs of multiple rock types. This paper presents the application of two state-of-the-art segmentation models, namely U-Net and LinkNet, to identify multiple rock types in digital photographs by segmenting the sandstone, mudstone, and background classes in a self-collected dataset of 102 images from a field in Brunei Darussalam. Four pre-trained networks, including *Resnet34*, *Inceptionv3*, *VGG16*, and *Efficientnetb7* were used as a backbone for both models, and the performances of the individual models and their ensembles were compared. We also investigated the impact of image enhancement and different color representations on the performances of these segmentation models. The experiment results of this study show that among the individual models, LinkNet with *Efficientnetb7* as a backbone had the best performance with a mean over intersection (MIoU) value of 0.8135 for all of the classes. While the ensemble of U-Net models (with all four backbones) performed slightly better than the LinkNet with *Efficientnetb7* did with an MIoU of 0.8201. When different color representations and image enhancements were explored, the best performance (MIoU = 0.8178) was noticed for the L*a*b* color representation with *Efficientnetb7* using U-Net segmentation. For the individual classes of interest (sandstone and mudstone), U-Net with *Efficientnetb7* was found to be the best model for the segmentation. Thus, this study presents the potential of semantic segmentation in automating the reservoir characterization process whereby we can extract the patches of interest from the rocks for much deeper study and modeling to be conducted.

## 1. Introduction

Rock type identification is a critical first step in resource exploration and development [1,2,3]. This involves a visual examination of the specimens for specific properties that are typically based on color, composition, sedimentary structures, and granularity [1]. Manual techniques are tedious, time-consuming, and can be subjective due to the quality of the preserved specimens [2].

Deep learning techniques have become highly popular and effective in solving real-world computer vision problems such as crack detection in concrete structures [4] and damage identification in building structures [5]. Automatic rock type identification using deep learning techniques is fast becoming popular due to its accuracy and efficiency [1,2,6]. Most of the existing image analysis approaches for rock classification consider one rock type per image. In reality, there can be many rock types within a single image.

Novel deep learning techniques have been applied to different types of rock images, such as microscopic imaging [6,7,8], computerized tomography (CT) [6,7], magnetic resonance imaging (MRI) [6,7], and digital camera photographs [1,2,9,10] for a qualification of the rock classification [1,2,6,7,10], granularity type [11], classification and measurement of absolute permeability, porosity, pore size distribution, n and ore distribution [9]. This study focuses on multi-rock type classifications using deep learning on digital camera photographs of field outcrops.

Ran et al. [1], Liang et al. [12] and Pascual et al. [10] applied deep learning frameworks to perform a single rock-type classification for each image that was sampled from the field rock images. Ran et al. [1] applied Random_Clip and Random_Flip operations on the images before feeding them into the CNN to classify the rock types in 2290 field rock photographs. Six rock types were classified, mylonite, granite, conglomerate, sandstone, shale and limestone. These images were cropped into 24,515 image patches. A mylonite classification achieved a 0-error rate, while the sandstone one had an error rate of 0.0406. Liang et al. [12] applied a framework comprising *cutmix*, *EfficientNetB0* and scoring by voting to classify the rock lithology of sandstone, mudstone, coal and siltstone and they achieved a 0.75 test accuracy. The pictures were taken both onsite and under white light in a dark box. The original image was segmented into 6 X 6 tiles, and these 36 tiles are of a lower resolution, and these pictures together construct a complete image. Each complete image contains one lithology. Pascual et al. [10] applied 3-layer Convolutional Neural Networks (CNN) on 700 textural images of nine different rock types, together with data augmentation, and they achieved a 0.99 test accuracy. Each image contains one lithology. Cheng and Guo [11] applied a CNN to classify the microscopic images of the rocks based on their granularity; coarse-, medium- or fine-grained.

Liu et al. [2] applied deep learning frameworks to perform both single and multiple rock-type classifications. They applied a faster R-CNN based on a simplified *VGG16* for feature extraction and rock type identification to be performed on 1034 rock images of eight types, basalt, conglomerate, gneiss, granite, limestone, magnetite quartz, marble and peridotite. The images were augmented and cropped, thereby increasing the sample size to 74,143. They tested single and multiple type rocks classifications, achieving accuracies of above 0.96 and 0.8, respectively. However, the sample images were a mixture of field images, extracted samples on a white background or they placed on a counter. Furthermore, to test their model for multi-rock type classification, they artificially merged the cropped image patches of the different rock types into one image and therefore, the test image of the different rock types is not a naturally occurring one. Interestingly, they trained the model on single rock-type samples and tested it on artificial multi-rock-type images.

Semantic segmentation using deep learning has been shown to effectively classify the images containing multiple classes, such as dermoscopy skin images containing multiple types of lesions [13] and classifying the brain injury lesions on CT images [14]. Semantic segmentation allows the labeling of specific regions of an image where multiple regions can belong to the same class, thereby making possible for multi-class segmentation to be performed [6,7]. The accurate segmentation of the rock images allows for the accurate identification of the rock types and descriptions of the geological materials. This is highly suitable for automating the identification of different rock types in one image.

Ringer and Yoon [6] applied semantic segmentation using four different CNN architectures, three 2D ones and one 3D one on X-ray computed microtomography (μCT) and focused ion beam-SEM (FIB-SEM) images to identify sandstone and shale rock types. For the 2D CNN classification, U-Net, U-*VGG16* and U-ResNet were used and for the 3D one, 3D U-Net was used. Images that were 128 × 128 were sampled from two 3D rock images, creating a total of 2569 segmented samples. The U-Resnet and U-VGG16 models achieved above 0.85 mean IOU scores. Alfarisi et al. [7] discussed the different types of machine learning algorithms that were applied on the CT and MRI scans of rocks to calculate the different properties that determine the rock types such as permeability and porosity. Jin et al. [9] developed a multi-module densely connected U-net to perform the semantic segmentation on digital camera photographic images of boreholes to determine the ore distribution and delineate the ore and waste rock boundary.

While Ran et al. [1], Pascual et al. [10] and Liang et al. [12] effectively applied deep learning techniques to classify the rock types in field rock images, their approaches could only handle a single rock-type classification per image. In our work, we apply deep learning to achieve a multi-rock-type classification. Liu et al. [2] applied deep learning techniques to classify multi-rock types on each image, however, the test was performed on artificially overlayed images of different rock types in a test sample and not on images of naturally occurring rock surfaces of multiple rock types. In our work, the model was trained and it was evaluated using images containing naturally occurring multi rock type surfaces. Semantic segmentation using deep learning was applied to classify the rock types by Ringer and Yoon [6], Alfarisi et al. [7] and Xu et al. [8], but these were on special images that were obtained from CT, MRI and SEM images, and not normal camera photographs. While Cheng and Guo [11] applied a CNN to classify the rock granularity and Niu et al. [15] applied a CNN on μCT and SEM images to segment the sandstone data and measure the properties relating to the rock type, these problems are not within the scope of this work. In our work, we applied semantic segmentation using deep learning techniques on digital photographs containing multiple rock types. Thus, our contribution is the application of semantic segmentation using U-Net and LinkNet to identify multiple rock types in digital photographs. Table 1 illustrates the types of deep learning rock type classification problems and their techniques and the research gap of this work.

In this work, we utilize high-resolution images that were acquired from an earlier outcrop study [16], which are dominated by sedimentary rocks. Here, the two main sedimentary rock types are sandstone beds and mudstone beds, which range in thickness from cm to dm. However, the majority of the beds are classified as thin-beds of less than 1 m. In the outcrop study, the individual beds have been measured, described, identified and interpreted, thereby adhering to the typical sedimentological study. Such a study requires significant amount of time and effort to acquire, analyze and understand the geology and its controlling factors. This forms an integral part of an analogue of the subsurface, of which the data availability and/or accessibility can be limited. Such an analogue can be used as proxy for some of the hydrocarbon fields that exist in NW Borneo as a means to interpret their geological significance and importance. The sedimentological study identified a total of 1483 sandstone beds with an equal number of mudstone beds over a length of 237 m stacked beds. The effort took five weeks for the geological observation to be conducted, which includes acquiring the high-resolution photos.

The aims of this study are: (1) to identify the two main rock types from a series of training images that were acquired from the outcrop study through the use of various deep learning techniques, and (2) to investigate the workflows that can be used as a tool for the rock type classifications from similar images and/or photographs.

In terms of the ongoing efforts towards the automation and decision support in reservoir characterization, we developed an automatic rock type classification system to identify the rocks of interest, in particular sandstone and mudstone. In this work, we aim to develop a multi-rock type classification system using deep learning techniques on digital outcrop rock images. To the best of our knowledge, there has been no study applying semantic segmentation for the multi-rock type classifications of field rock images. This is a preliminary step towards the sedimentological characterization of the facies that have been identified. Using semantic segmentation to classify sandstone and mudstone, we can quickly extract the rock patches of interest for a much deeper study and modelling to be conducted, such as calculating the rock distribution [9].

**Figure 1:**
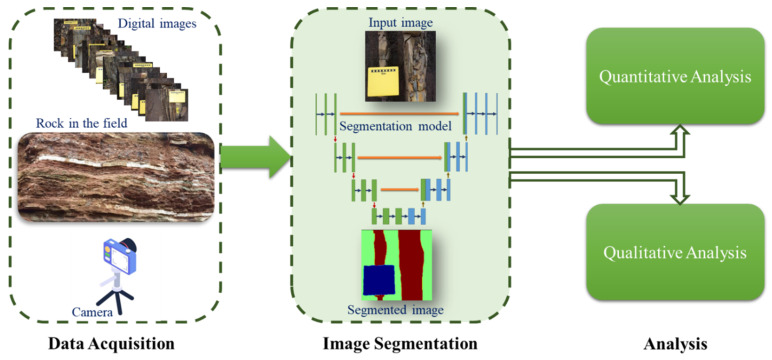
Overall flow of the proposed system.

In this work, two different models, U-Net and LinkNet, were applied for the segmentation of the sandstone and mudstone rocks with various encoder backbones, *Efficientb7*, *Resnet34*, *Inceptionv3* and *VGG16* [17,18], and ensemble learning, and their results were analyzed both quantitatively and qualitatively (Figure 1).

In Section 2, we explain the methodology, as briefly explained above, which was applied to perform the sandstone and mudstone rock segmentation of the digital outcrop photos. In Section 3, we present, interpret and discuss the results that were obtained from our investigations. Finally, we summarize our work and share the key lessons in the conclusion section.

## 2. Materials and Methods

**Figure 2:**
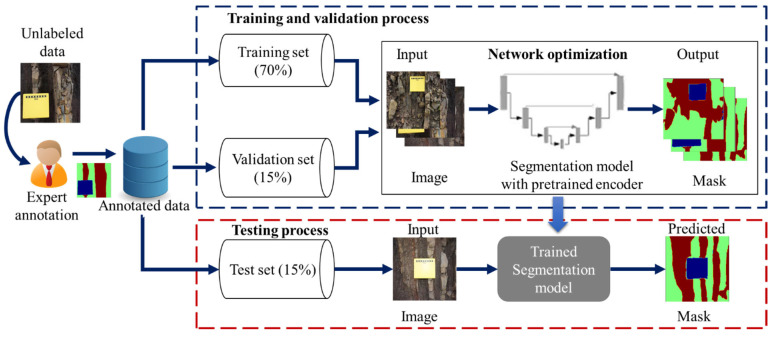
Experimental framework.

The proposed methodology that was used in this study is depicted in Figure 2. The details of each step are explained in the following sections.

### 2.1. Dataset Collection and Annotation

**Figure 3:**
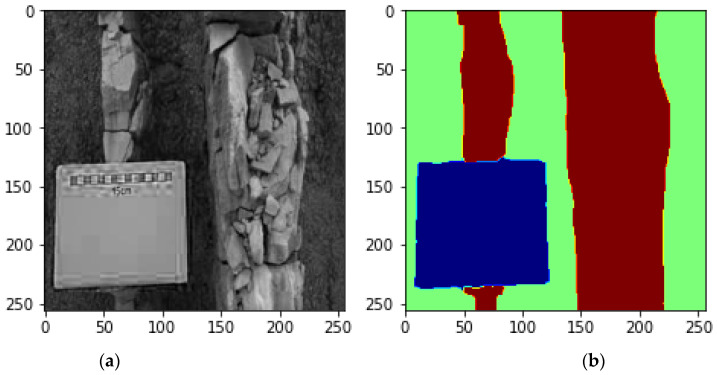
An example of (**a**) an original image and (**b**) its labels—background (blue), mudstone (wine) and sandstone (green).

A total of 102 images which were used in this study were collected using a high-resolution digital camera. Each image consisted of multiple instances of sandstone and mudstone data. The resolution of the images was 6000 × 4000 pixels. The images were annotated by a field expert to generate a semantic segmentation mask to train the deep learning models. Each image pixel was assigned a label to represent either a pixel belonging to a mudstone class or a sandstone class or the background class (Figure 3). Thus, there were 375 instances of sandstone, 312 instances of mudstone and 138 background instances which were labeled for the training, validation and testing of the semantic segmentation models. The APEER (https://www.apeer.com/, accessed on 24 January 2022) annotation tool was used to annotate the dataset and generate the ground truth labels.

### 2.2. Pre-Processing

Together with their ground truth annotations, all of the input images were resized to 256 × 256 pixels to reduce the computational complexity in processing the higher resolution images. A standard preprocessing step was then applied to maximize the performance gain based on the backbone encoder. For example, when we were using *Resnet* as an encoder backbone, all of the input images were first converted from an RGB to an BGR color channel and then, we zero-centered each color channel based on a specific ImageNet dataset without pixel scaling it. On the other hand, the backbone encoders such as *Efficientb7* did not require any pixel scaling as the model consists of a rescaling layer that automatically scales the input images during the model training and inference.

The color transformations and image enhancement (e.g., histogram equalization) have been found to be helpful in improving the classification and segmentation of the images in previous studies [19,20,21]. We evaluated the effect of the histogram equalization and color transformations to RGB images on the segmentation performance. To apply the histogram equalization on the multichannel color image (i.e., RGB), the image was converted to a YCrCb format, and then, the histogram equalization was applied to the luminance channel. The training/testing dataset was generated by converting back the output image to the RGB format. The impact of the color transformations was also investigated by using three common color-space models including YCrCb, L*a*b* and HSV.

### 2.3. Segmentation Models

Two different semantic segmentation networks were compared to assess the extent of their performance when they were used for the segmentation of the sandstone and mudstone rocks with various encoder backbones. These models have achieved state-of-the-art results in different segmentation tasks with varying degrees of complexity [22]. The summary of these models is explained below.

U-Net: U-Net was first proposed for performing segmentation tasks in the biomedical domain [23]. Due to its performance and efficiency with smaller datasets, it has now been adapted in different domains. Like the other semantic segmentation networks, U-Net follows an encoder–decoder architecture where an encoder down-samples the image to capture the context, while a decoder part of the network symmetrically expands to enable the precise localization of the objects in an image. A 3 × 3 convolution extracts the features while down-sampling the image, and a de-convolution process is then applied to up-sample the feature map. Skip connections are added to the encoder and the decoder parts to copy the feature maps to avoid the occurrence of information loss. Finally, a 1 × 1 convolution is applied to generate a segmentation mask by classifying each pixel into a specific class.

LinkNet: LinkNet architecture was proposed with the aim of improving the information sharing between the encoder part and the decoder part [24]. This was achieved by replacing the convolution layers of the U-Net architecture with the *Resnet* blocks on the encoder and decoder parts. In addition, LinkNet uses the addition method instead of the stacking method (present in U-Net) to transform the synthesis features.

These models follow a generic encoder–decoder structure where the encoder extracts the key features from the input image and then uses the decoder part to project the features into the pixel space to obtain a pixel-level classification. The encoder part is usually a pre-trained model, which is used as a backbone encoder, to improve the network learning speed and performance by taking advantage of the good weight initialization. In this study, we assessed the performance of the U-Net and LinkNet models on four widely used classification networks. These models include *VGG16*, *Resnet34*, *Inceptionv3* and *Efficientb7,* and they have been widely adopted as the backbone feature extractors for various computer vision tasks [17,18]. While using a pre-trained network as an encoder, the normal convolution layers are replaced with the backbone module layers. For example, the convolution layers in a standard U-Net architecture are replaced with the *Resnet* blocks when one is using *Resnet* as the network backbone encoder. Similarly, the *Resnet* modules are adopted in the decoder part to replace the deconvolution layers.

### 2.4. Network Training

For both U-Net and LinkNet, a batch size of 4 images was used with an Adam optimizer with a learning rate of 0.0001. All of the backbone architectures were initialized with the ImageNet weights since it has been repeatedly shown that initializing a network with the trained weights helps speed up the training and network convergence [25]. An early stopping criterion was used to find the best-performing model within the first 100 training epochs by monitoring the validation loss. All of the networks were trained with a combination of focal loss and dice loss, having a class weight of 0.2 for the background class, a class weight of 0.4 for the Mudstone class and a class weight of 0.4 for the Sandstone class. This approach was used to encourage the models to perform better on segmentation of the Mudstone and Sandstone classes.

All of the experiments were conducted using the TensorFlow deep learning framework on a machine that was equipped with an Intel Core i7 and NVIDIA GeForce RTX 3070 with 32 GB RAM.

### 2.5. Ensemble Predictions

We also investigated the use of an ensemble strategy to improve the performance of the predicted output mask. Ensemble learning is commonly used to combine the performance of weak models to make final predictions [26]. When one is performing a model ensemble, the results of each model prediction are aggregated via a weighted average on a test set. We adopted the weighted average ensemble prediction technique from the individual models to make the final predicted mask. All of the models were equally weighted to contribute to the ensemble prediction.

### 2.6. Performance Evaluation

In this study, we evaluated the performance of the models using mean intersection over union (MIoU), which is a widely adopted metric for measuring the performance of the segmentation models [27]. The MIoU calculates an average score of all of the classes (background, mudstone, and sandstone) by measuring the overlap between the target and predicted classes. This is achieved by finding the ratio of the true positive over the sum of true positive, false positive and false negative of the segmented pixels as calculated below:

(1) $$\mathrm{MIoU}\;=\frac{1}{N_{cls}}\overset{N_{cls}}{\underset{x=1}{\sum }}\;\frac{N_{xx}}{\sum_{y=1}^{N_{cls}}\;N_{xy}+\sum_{y=1}^{N_{cls}}\;N_{yx}-N_{xx}}$$

where $N_{xx}$ is the true positive pixels, $N_{cls}$ is the total number of classes, $N_{yx}$ is the false negative pixels and $N_{xy}$ is the false positive pixels. In this study, we used a threshold of 0.5 overlap to determine the IoU score of the model predictions. Further, the precision (2), recall (3) and F1-score (4) were also computed to observe the performances of the different models.

(2) $$\mathrm{Precision}\;=\frac{N_{xx}}{N_{xx}+N_{xy}}$$

(3) $$\mathrm{Recall}\;=\frac{N_{xx}}{N_{xx}+N_{yx}}$$

(4) $$F1-\mathrm{score}\;=\frac{2\times \mathrm{Precision}\;\times \mathrm{Recall}}{\mathrm{Precision}+\mathrm{Recall}}$$

## 3. Results and Discussion

A total of 102 images were used, with 70% of the data being used for training, 15% of it being used for validation and 15% of it being used as a test set. Each model was optimized to reflect the best performance by tuning the model hyperparameters to the training set. The MIoU was used to evaluate the performance of each model. Table 2 and Table 3 show the performance comparison between the U-Net and LinkNet models with different backbone encoders based on the MIoU metric.

**Figure 4:**
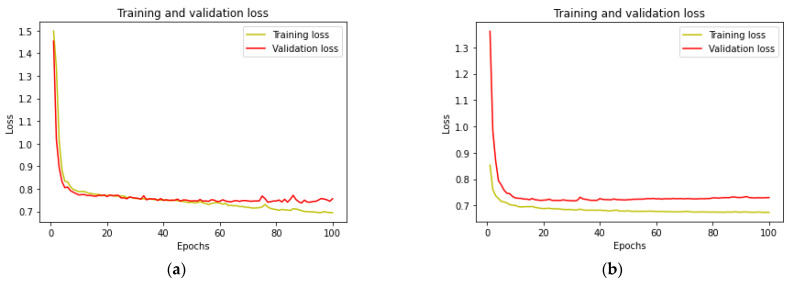
Training and validation loss curve for a Standard U-Net model (**a**) vs. U-Net with *Efficientb7* encoder (**b**).

When observing the performance of a U-Net architecture, the use of a pretrained backbone feature extractor has a clear advantage as it significantly improves the model convergence and performance over the vanilla architecture. This is evident when looking at the training and validation loss graphs for both the vanilla U-Net and the one using the backbone extractor in Figure 2. Using vanilla/standard U-Net without any backbone feature extractor, the network struggles to converge until around 90 epochs. On the other hand, using a pretrained backbone enables the network to converge faster even when it is using such a small training dataset, as seen in Figure 4.

### 3.1. Individual Models vs. Ensemble

From the results in Table 2 and Table 3, we can observe that using the ensemble learning strategy resulted in slightly higher performance gains than the individual models obtained, with the exception of the *Efficientb7*-based models. For example, with the use of ensemble learning in the U-Net model, an MIoU of 0.8201 was achieved, which is higher than those which were achieved by the other backbone encoders such as *Efficientb7*, *Resnet34*, *Inceptionv3* and *VGG16*. This performance is comparable when it we were using either of the two segmentation models.

When looking at the individual class performance, a similar pattern can be observed in both ensemble and individual models, with there being a higher performance in the segmentation of the background class than in the segmentation of mudstone and sandstone. However, an ensemble of U-Net models shows a slightly higher performance for the mudstone and sandstone classes on a test set with MIoUs of 0.7726 and 0.8003, respectively, which are almost 2% better than the best-performing U-Net *Efficientb7* model. This suggests that the ensemble learning strategy offers a better performance when one is accessing the performance of the individual classes and the overall model performance.

**Figure 5:**
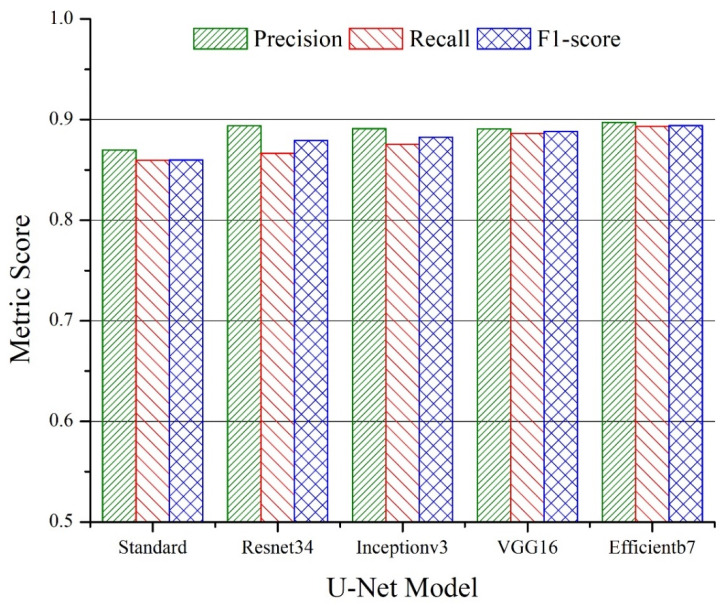
Average values of precision, recall and F1-score for U-Net models with different backbones for test data.

**Figure 6:**
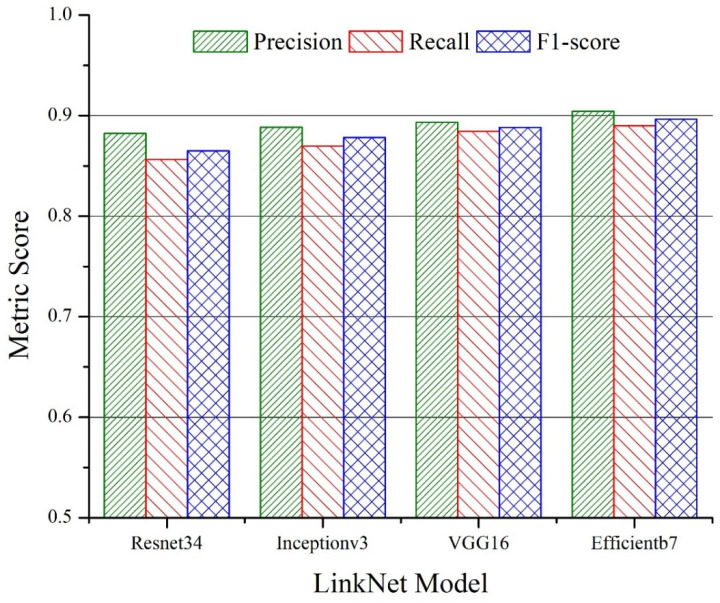
Average values of precision, recall and F1-score for LinkNet models with different backbones for test data.

The average values of precision, recall and the F1-score are plotted in Figure 5 and Figure 6 for the U-Net and LinkNet models with different backbones, respectively. The superior performance of the *Efficientb7*-based backbone can be observed for both U-Net and LinkNet segmentation models. A higher value of the precision metric was noticed for all of the backbones for both models indicating that more relevant pixels were correctly retrieved compared to number of the irrelevant pixels. Since the goal of the segmentation models was to retrieve the sandstone class more accurately, we further analyzed the performance of the models at the individual levels. For the sandstone class, the overall best performance was observed for the LinkNet model with the *Efficientb7* backbone with a precision, recall and F1-score of 0.9001, 0.8685 and 0.8840, respectively. A slightly better value of precision (0.9103) was seen for the U-Net model with the *Efficientb7* backbone in the sandstone class with a slightly low value of recall (0.8500).

### 3.2. U-Net vs. LinkNet

The U-Net and LinkNet models had a matching performance on both the validation and test sets (Table 2 and Table 3). Despite the LinkNet architecture having a Resnet-inspired encoder structure, the U-Net model with the *Resnet34* encoder provided a better performance than LinkNet did, with it having an MIoU of 0.7854 on a test set over an MIoU of 0.7634 for the LinkNet with the *Resnet34* encoder. This may also be attributed to it having a slightly higher number of trainable parameters than the LinkNet models did. However, by looking at the overall performance of both model variants, this suggests that the LinkNet model also performed better on the individual classes. For example, when looking at the performance of LinkNet with the *Efficientb7* encoder, this model achieved an MIoU of 0.7922 in the segmentation of the sandstone class, which is slightly higher than using the *Efficientb7* encoder achieved in a U-Net architecture. The results also suggest that the performance between the two architectures varied depending on the backbone encoder that was used.

### 3.3. Comparison of Different Backbone Architecture

Each of the backbone architectures that were applied present unique features for the segmentation network. Based on the results, it is clear that using the *Efficientb7* as a backbone feature encoder enabled the segmentation network to generate more discriminating features than the other backbone encoders could with a higher number of parameters. Both the U-Net and LinkNet architectures outperformed the other models when they were using *Efficientb7* as a backbone encoder on validation and test set. On the other hand, the remaining backbone architectures such as *VGG16*, *Resnet34* and *Inceptionv3* had a matching performance. We can also notice that *VGG16* as an encoder outperformed both *Resnet34* and *Inceptionv3*, despite it having few model parameters on the test set. It is likely that the simple stacking of the convolution layers in *VGG16* which served as the encoder part can extract meaningful features of the images. On the other hand, since both sandstone and mudstone appeared in almost equal proportion relative to image size, a less complex network such as *VGG16* can be an effective encoder.

When looking at the individual class performance of the backbone encoders, the performance of the background class outperformed both the sandstone and mudstone segmentation. This suggests that regardless of the encoder architecture that was used, all of the models struggled to segment the classes other than the background, as the background class mainly consisted of a uniform appearance in almost all of the images. On the other hand, all of the models struggled to perform more in segmentation in the mudstone class than they did in the sandstone class. The sandstone class likely occupied a large proportion of the images compared to the mudstone class.

### 3.4. Effect of Image Enhancement and Color Transformations

**Figure 7:**
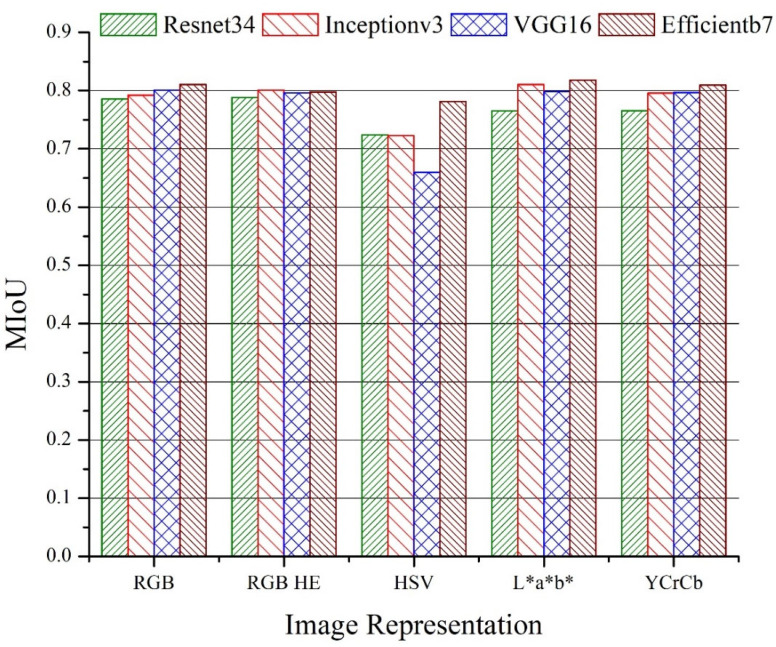
Mean intersection of union (MIoU) for U-Net models with different backbones and image color representations for test data.

**Figure 8:**
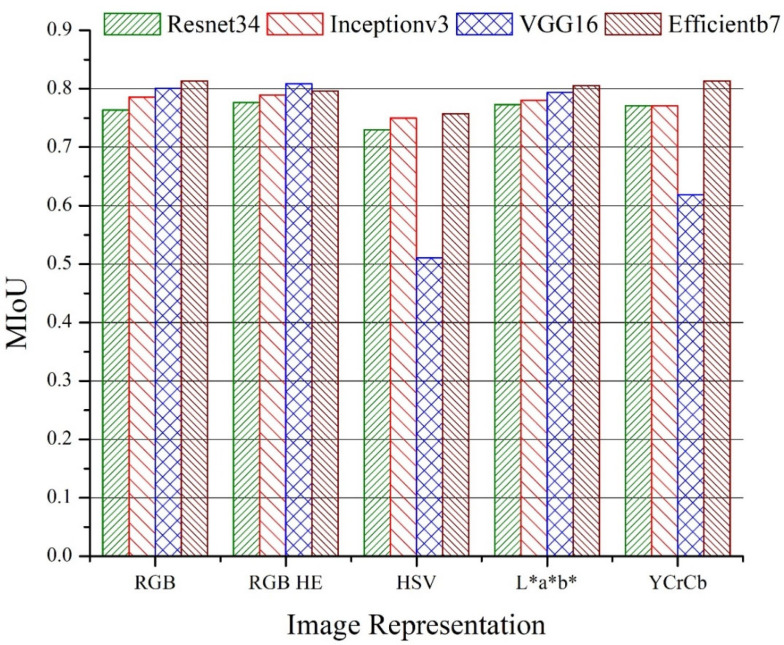
Mean intersection of union (MIoU) for LinkNet models with different backbones and image color representations for test data.

The effect of histogram equalization and color transformation to RGB images on the performance of segmentation was also analyzed. Figure 7 and Figure 8 represent the MIoUs for the U-Net and LinkNet models, respectively, with various backbone networks and different color representations. The best performance (MIoU = 0.8178) was noticed for the L*a*b* color representation with *Efficientb7* using U-Net segmentation, which was slightly higher than the MIoU of the RGB color representation (0.8102) with the same model and backbone. More than 2% improvement was noticed in the performance of the U-Net model with the *Inceptionv3* backbone for the L*a*b* color representation as compared to that of the RGB color representation. There was not much significant improvement which was observed for the histogram equalization over the RGB representation on the performance of both the U-Net and LinkNet models. We also found that the HSV color representation resulted in a poor performance for the segmentation of the three classes, especially for LinkNet with the *VGG16* backbone. In general, the performance did not vary too much for all of the representations except for the models that were based on the HSV color representation.

The intersection of union (IoU) of the individual classes for the different color representations for the U-Net and LinkNet models are presented in Table 4 and Table 5, respectively. The IoU for the sandstone class varies between 0.6762 (HSV with *Inceptionv3*) and 0.8012 (L*a*b* with *Efficientb7*) for the U-Net segmentation models. For most of the backbones with the U-Net model, the IoU value for the sandstone class is above 0.7. In contrast to the U-Net model, the best value of the IoU for sandstone was 0.7998 for the LinkNet model with the YCrCb color model and the *Efficientb7* backbone network. Again, a poor performance was noticed for the HSV color representation, especially with the *VGG16* backbone network. Overall, *Efficientb7* was observed as the best individual backbone for both the U-Net and LinkNet models.

### 3.5. Qualitative Analysis of Segmentation Models

**Figure 9:**
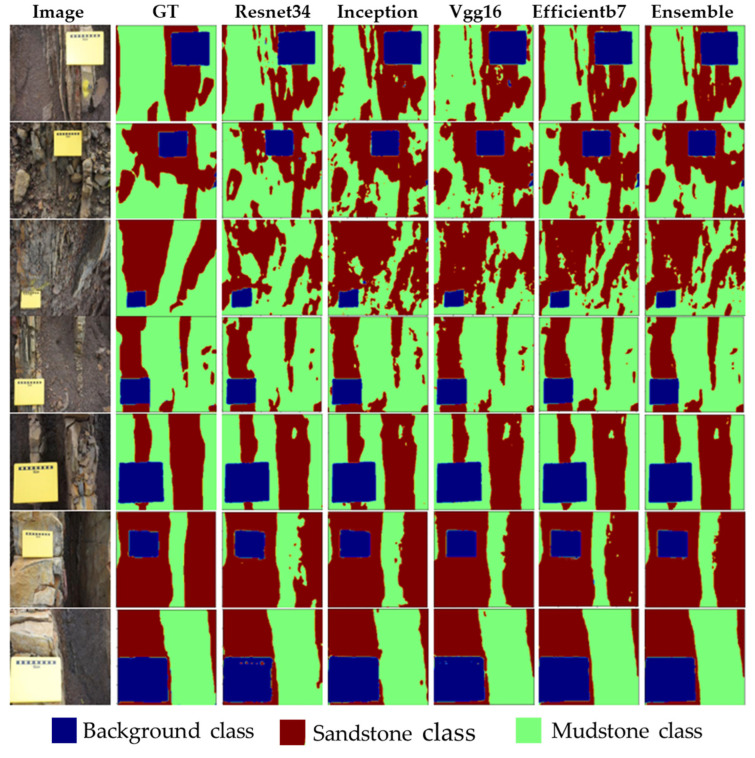
Prediction results for LinkNet model with different backbones.

**Figure 10:**
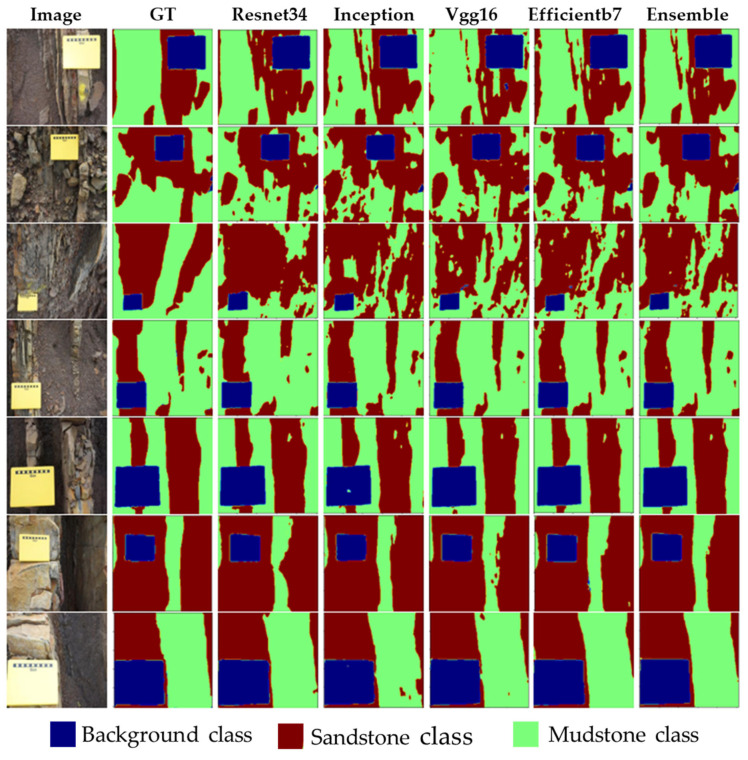
Prediction results for U-Net model with different backbones on a test set.

Figure 9 and Figure 10 present the comparison of the predicted masks for the different backbone encoders on selected images from the test set. The visual inspection of the output suggests that for the wider and more intact sandstone regions, the models performed better, while for a mix of sandstone and mudstone or for broken, small pieces of sandstone, the performance of the models was low. For example, when looking at the image of row three in Figure 9 and Figure 10, most of the mudstone class has been misclassified as a sandstone class. In contrast, rows five to seven show a better segmentation performance for all of the models for the wide and intact sandstones. In general, all of the models tended to perform better on the segmentation of the background class than they did for the other classes. Though the error is observable in Figure 9 and Figure 10, the precision analysis that is presented in Section 3.1 provides more insight into the efficacy of the semantic segmentation models. For the sandstone class, higher values of the precision and recall scores were noticed for both the LinkNet and U-Net models with the *Efficientb7* backbone. An important point to note is that the intact and wider sandstones are of more interest to the domain experts for the reservoir/rock characterization. The overall objective of applying semantic segmentation for the given data set is to roughly estimate the amount of sandstone that is present in the rock/reservoir of interest. The percentage of the sandstone for an individual image can be computed using semantic segmentation and for the whole data set (consisting of *n* images), the total percentage of sandstone that is present in the rock can be computed by combining these individual results.

## 4. Conclusions and Future Work

This study assessed the extent of using machine learning to segment the sandstone and mudstone classes using the digital images that were collected from the field. Two existing state-of-the-art models, including U-Net and LinkNet, were compared with different backbone encoders. The results suggest that these models can obtain a reasonable performance when they are segmenting between sandstone and mudstone rocks even with a small training dataset. On the other hand, using pretraining encoders as network backbones can further improve their performance. Specifically, a backbone encoder such as *Efficientb7* offers a more performance advantage than the other encoders do such as *Resnet34*, *VGG16* and *Inceptionv3*. Finally, using an ensemble of models improved the model’s performance both when assessing the individual class performance and the overall MIoU. There were not many significant improvements that were noticed in the performance of the segmentation models after performing the image enhancement (histogram equalization) and changing the color representations from RGB to other color types.

Based on the result of this study, the future work will focus on designing a more efficient encoder backbone to work with a small training sample. Other techniques such as few-shot learning for semantic segmentation will be explored to reduce the reliance on a large training dataset. To increase the confidence in rock identification for sandstone, there is an opportunity to incorporate sedimentary structures as part of the overall machine learning workflow.

To extend the automation capability of multi-rock type identification, we will investigate our existing deep learning framework and extend its capability to quantify the proportion of the different rock types that were found in the digital photographs. The automation of such process will assist geologists in processing, identifying and quantifying the different rock types in large volumes of photographs. This will involve measuring the segmented areas of interest relating to the rock types and evaluating them against the human-measured proportions.

## Figures and Tables

**Table 1:** Different types of deep learning rock type classification problems and solutions.

Literature	Type of Images	(S)ingle or (M)ultiple Rock Type Per Image	Techniques	Semantic Segmentation
Ran et al. [1]	digital camera photographs	S	CNN	N
Liu et al. [2]	digital camera photographs	M ^1^	faster R-CNN, simplified *VGG16*	N
Ringer and Yoon [6]	SEM, CT	S	U-Net, U-*VGG16*, U-*Resnet*	Y
Alfarisi et al. [7]	SEM, CT, MRI	S	ML, CNN	Y ^2^
Xu et al. [8]	polarizing microscope	S	*Xception*, *MobileNetv2*, *Inception_Resnetv2*, *Inceptionv3*, Densenet121, *Resnet101v2*, and *Resnet101*	N
Pascual et al. [10]	digital camera photographs	S	3-layer CNN, Transfer Learning	N
Cheng and Guo [11]	polarized light microscopy	S	CNN	N
Liang et al. [12]	digital camera photographs	S	*EfficientNetB0*	N
Niu et al. [15]	μCT, SEM	S	*LeNet5*	N
Ours	digital camera photographs	M	U-Net, LinkNet, Transfer learning, Ensemble learning	Y

^1^ artificially overlaid; ^2^ discussed only.

**Table 2:** Performance evaluation of U-Net models for validation and test sets. The best test results are presented in bold.

	Validation Set				Testing Set			
Network	Background	Mudstone	Sandstone	MIoU	Background	Mudstone	Sandstone	MIoU
U-Net Standard	0.9189	0.6869	0.6774	0.7611	0.8594	0.7092	0.7027	0.7571
U-Net *Resnet34*	0.9257	0.7566	0.7850	0.8225	0.8475	0.7353	0.7735	0.7854
U-Net *Inceptionv3*	0.9257	0.7140	0.7740	0.8046	0.8768	0.7266	0.7724	0.7919
U-Net *VGG16*	0.9288	0.7557	0.7868	0.8238	0.8798	0.7464	0.7754	0.8005
U-Net *Efficientb7*	0.9333	0.7983	0.8204	0.8507	0.8848	0.7615	0.7843	0.8102
U-Net Ensemble	0.9381	0.7902	0.8222	0.8502	**0.8873**	**0.7726**	**0.8003**	**0.8201**

**Table 3:** Performance evaluation of LinkNet models for validation and test sets. The best test results are presented in bold.

	Validation Set				Testing Set			
Network	Background	Mudstone	Sandstone	MIoU	Background	Mudstone	Sandstone	MIoU
LinkNet *Resnet34*	0.9180	0.7426	0.7422	0.8009	0.8328	0.7223	0.7351	0.7634
LinkNet *Inceptionv3*	0.9287	0.7431	0.7826	0.8181	0.8721	0.7165	0.7671	0.7852
LinkNet *VGG16*	0.9322	0.7411	0.7636	0.8123	0.8855	0.7432	0.7734	0.8007
LinkNet *Efficientb7*	**0.9393**	**0.8064**	**0.8331**	**0.8596**	0.8840	0.7643	0.7922	0.8135
LinkNet Ensemble	0.9390	0.8028	0.8246	0.8554	0.8851	0.7672	0.7961	0.8161

**Table 4:** Performance evaluation (IoU) of U-Net models for individual classes in test sets using different image color representations. The best model performance for each class is presented in bold.

Backbone	Class	RGB	RGB HE	HSV	L*a*b*	YCrCb
*Resnet34*	Background	0.8475	0.8454	0.8382	0.8674	0.8654
	Mudstone	0.7353	0.7439	0.6346	0.7119	0.7163
	Sandstone	0.7735	0.7735	0.6985	0.7164	0.7156
*Inceptionv3*	Background	0.8768	0.8711	0.8504	0.8771	0.8674
	Mudstone	0.7266	0.7518	0.6417	0.7624	0.7413
	Sandstone	0.7724	0.7786	0.6762	0.7921	0.7779
*VGG16*	Background	0.8798	0.8631	0.6050	**0.8832**	0.8808
	Mudstone	0.7464	0.7490	0.6814	0.7370	0.7308
	Sandstone	0.7754	0.7767	0.6923	0.7743	0.7784
*Efficientb7*	Background	0.8848	0.8840	0.8596	0.8800	0.8774
	Mudstone	0.7615	0.7516	0.7318	**0.7722**	0.7567
	Sandstone	0.7843	0.7563	0.7536	**0.8012**	0.7933

**Table 5:** Performance evaluation (IoU) of LinkNet models for individual classes in test sets using different image color representations. The best model performance for each class is presented in bold.

Backbone	Class	RGB	RGB HE	HSV	L*a*b*	YCrCb
*Resnet34*	Background	0.8328	0.8249	0.8313	0.8663	0.8643
	Mudstone	0.7223	0.7447	0.6581	0.7101	0.7039
	Sandstone	0.7351	0.7600	0.7002	0.7420	0.7438
*Inceptionv3*	Background	0.8721	0.8725	0.8504	0.8690	0.8457
	Mudstone	0.7165	0.7357	0.6832	0.7182	0.7074
	Sandstone	0.7671	0.7586	0.7161	0.7545	0.7593
*VGG16*	Background	0.8855	**0.9258**	0.8253	0.8797	0.8798
	Mudstone	0.7432	0.7399	0.1454	0.7293	0.3446
	Sandstone	0.7734	0.7593	0.5618	0.7713	0.6319
*Efficientb7*	Background	0.8840	0.8643	0.8480	0.8785	0.8783
	Mudstone	**0.7643**	0.7531	0.6908	0.7490	0.7618
	Sandstone	0.7922	0.7711	0.7336	0.7882	**0.7998**

## Data Availability

These data were 3rd Party Data. Data were obtained from I.P and they are privately owned.
